# Excellent Temperature Performance of Spherical LiFePO_4_/C Composites Modified with Composite Carbon and Metal Oxides

**DOI:** 10.1155/2014/364327

**Published:** 2014-01-09

**Authors:** Bao Zhang, Tao Zeng, Jiafeng Zhang, Chunli Peng, Junchao Zheng, Guomin Chen

**Affiliations:** School of Metallurgy and Environment, Central South University, Changsha, Hunan 410083, China

## Abstract

Nanosized spherical LiFePO_4_/C composite was synthesized from nanosized spherical FePO_4_ *·*2H_2_O, Li_2_C_2_O_4_, aluminum oxide, titanium oxide, oxalic acid, and sucrose by binary sintering process. The phases and morphologies of LiFePO_4_/C were characterized using SEM, TEM, CV, EIS, EDS, and EDX as well as charging and discharging measurements. The results showed that the as-prepared LiFePO_4_/C composite with good conductive webs from nanosized spherical FePO_4_ *·*2H_2_O exhibits excellent electrochemical performances, delivering an initial discharge capacity of 161.7 mAh*·*g^−1^ at a 0.1 C rate, 152.4 mAh*·*g^−1^ at a 1 C rate and 131.7 mAh*·*g^−1^ at a 5 C rate, and the capacity retention of 99.1%, 98.7%, and 95.8%, respectively, after 50 cycles. Meanwhile, the high and low temperature performance is excellent for 18650 battery, maintaining capacity retention of 101.7%, 95.0%, 88.3%, and 79.3% at 55°C, 0°C, −10°C, and −20°C by comparison withthat of room temperature (25°C) at the 0.5 C rate over a voltage range of 2.2 V to 3.6 V, respectively.

## 1. Introduction

Clean and rechargeable secondary energy sources are urgently needed for the development of modern technology and widespread application of portable electrical equipment. Among secondary energy sources, rechargeable lithium- (Li) ion battery is the most attractive and promising. In 1997, Padhi et al. [1] proved that olivine LiFePO_4_ has an excellent performance in Li-ion intercalation and deintercalation. Its theoretical capacity is 170 mAh·g^−1^ with a flat discharge voltage at 3.4 V. Among polyanion cathodes, LiFePO_4_ has the advantages of excellent cycle ability, good thermal stability, inexpensive raw materials, and environmental friendliness. LiFePO_4_ is also attractive for use as a next generation cathode material for Li-ion batteries. However, the low electronic conductivity of LiFePO_4_ leads to a poor charge-discharge performance at a high current rate. Therefore, modifications to LiFePO_4_ to improve its properties have been developed. Such modifications include coating with nanocarbon [2–9], encapsulation with a conductive polymer [10, 11], and doping with suitable metals [12–18].

FePO_4_·*x*H_2_O is a promising precursor for preparing the Li-ion battery cathode material LiFePO_4_. The advantages of this precursor include innocuity, low cost, similar structure with LiFePO_4_, and oxidation avoidance of ferrous iron [19–21]. Traditional methods for the synthesis of FePO_4_·*x*H_2_O and LiFePO_4_ include solid phase synthesis [22, 23], sonochemistry [24], coprecipitations [25–29], and hydrothermal synthesis [30–32]. These methods usually involve the use of expensive metal-organic compounds. In the solid-state synthetic process, high-energy consumption is generated and particles are relatively unevenly distributed. The sonochemistry process has some advantages in preparing iron phosphate, including nonrequirement of oxidant, less reaction time, and controllable particle size. However, the large-scale production of iron phosphate is difficult to be realized [33, 34]. Lee and Teja [35] Xu et al. [36] prepared LiFePO_4_ nanoparticles in subcritical and superheated water. Yu et al. [37] investigated the rapid and continuous production of LiFePO_4_/C nanoparticles in superheated water. Chen et al. [38] reported the influences of carbon sources on the electrochemical performances of LiFePO_4_/C composites.

In this work, nanosized spherical LiFePO_4_/C composite was prepared from nanosized spherical FePO_4_·2H_2_O, Li_2_C_2_O_4_, aluminum oxide, titanium oxide, oxalic acid, and sucrose by binary sintering process. The process is simple, requires uncomplicated equipment, and consumes low energy. Fine particles were obtained in homogenous distribution, appropriate for industrialized production.

## 2. Experimental

### 2.1. Preparation of Materials

The LiFePO_4_/C composite was prepared by mixing stoichiometric amounts of nanosized spherical FePO_4_·2H_2_O, Li_2_C_2_O_4_, aluminum oxide, and titanium oxide dispersed in ethylene glycol with oxalic acid and sucrose, followed by grinding via ball milling. After evaporating the ethylene glycol, the mixture was firstly sintered in a horizontal quartz tube at 400°C for 6 h in an argon atmosphere. As the presintered product cooled to room temperature, the LiFePO_4_/C composites were obtained after being calcined at 650°C for 8 h.

### 2.2. Material Characterization

The LiFePO_4_/C sample was characterized by X-ray diffraction (XRD; Rigaka D/MAX 2500 V) using CuK*α* radiation (K*α* = 0.154 nm) to identify the crystalline phase. Data were collected between 10° and 90° in steps of 8°/min. The surface morphologies of the samples were observed by a scanning electron microscopy (SEM) system (JSM-6380LV). The details of carbon coating were observed with transmission electron microscopy (TEM) (Hitachi, H-8100). A three-electrode system was used to characterize the electrochemical performances of the as-deposited LiFePO_4_/C composites. LiFePO_4_/C freshly deposited under the same experimental parameters were used in cyclic voltammetry (CV) analysis and normal charging and discharging experiments, as well as high rate charge and discharge characterizations. Electrochemical impedance spectroscopy (EIS) measurements were performed over a frequency range of 0.001 Hz to 1 MHz at a 50% discharge stage with a perturbation signal of 5 mV over a Chi 660c setup. All electrochemical measurements were conducted at room temperature (25°C).

### 2.3. Electrode Fabrication and Electrochemical Measurements

The as-prepared cathode was mixed with acetylene black and polyvinylidene difluoride at a mass ratio of 80 : 10 : 10. LiFePO_4_/C cathode was prepared by spreading the above mixture on an aluminum foil and drying in a vacuum oven at 120°C. Charge-discharge tests on LiFePO_4_/C were performed in coin cells using LiFePO_4_/C cathodes and Li anodes. A porous membrane (Celgard 2300) was used as a separator, and the electrolyte was 1 mol·L^−1^ LiPF_6_ dissolved in a mixture of ethylene carbonate, dimethyl carbonate, and methyl-ethyl carbonate at a volume ratio of 1 : 1 : 1. Coin cells (CR 2025) were assembled in an argon-filled glove box. The cells were charged and discharged at the rates of 0.1 C, 1 C and 5 C over a voltage range of 2.5 V to 4.2 V, respectively, versus the Li/Li^+^ electrode at ambient temperature using a battery testing system (Neware BTS-2000). The high and low temperature performance of LiFePO_4_/C was tested via fabricating 18650 battery at the rate of 0.5 C over a voltage range of 2.2 V to 3.6 V.

## 3. Results and Discussion

### 3.1. Morphology of LiFePO_**4**_/C Prepared with Nanosized Spherical FePO_**4**_·2H_**2**_O

**Figure 1:**
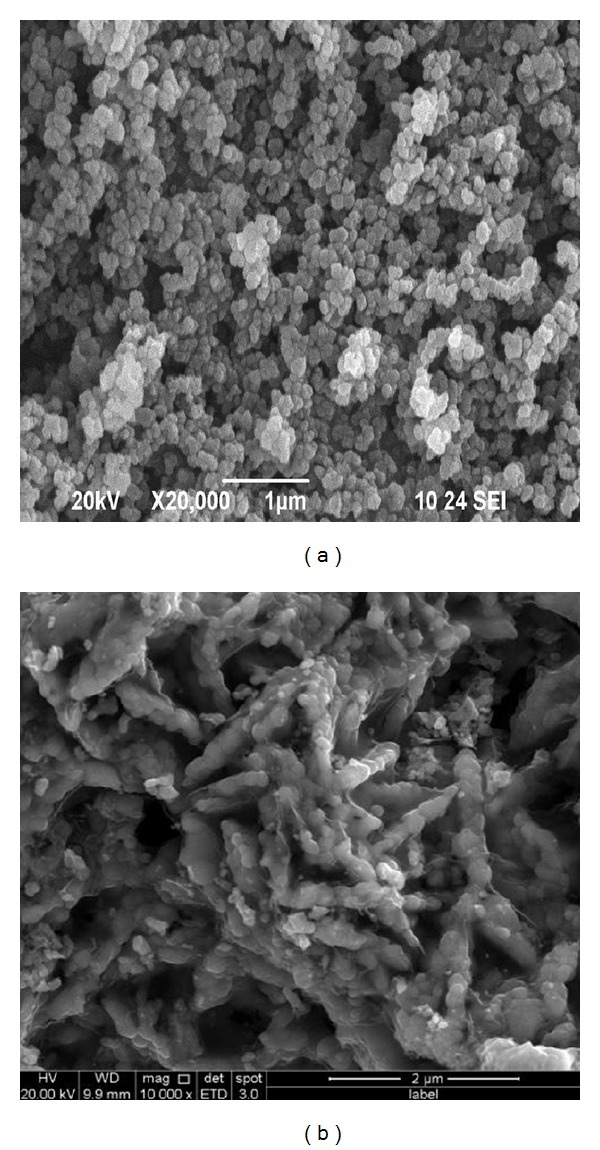
SEM images of nanosized spherical FePO_4_·2H_2_O and LiFePO_4_/C.

**Figure 2:**
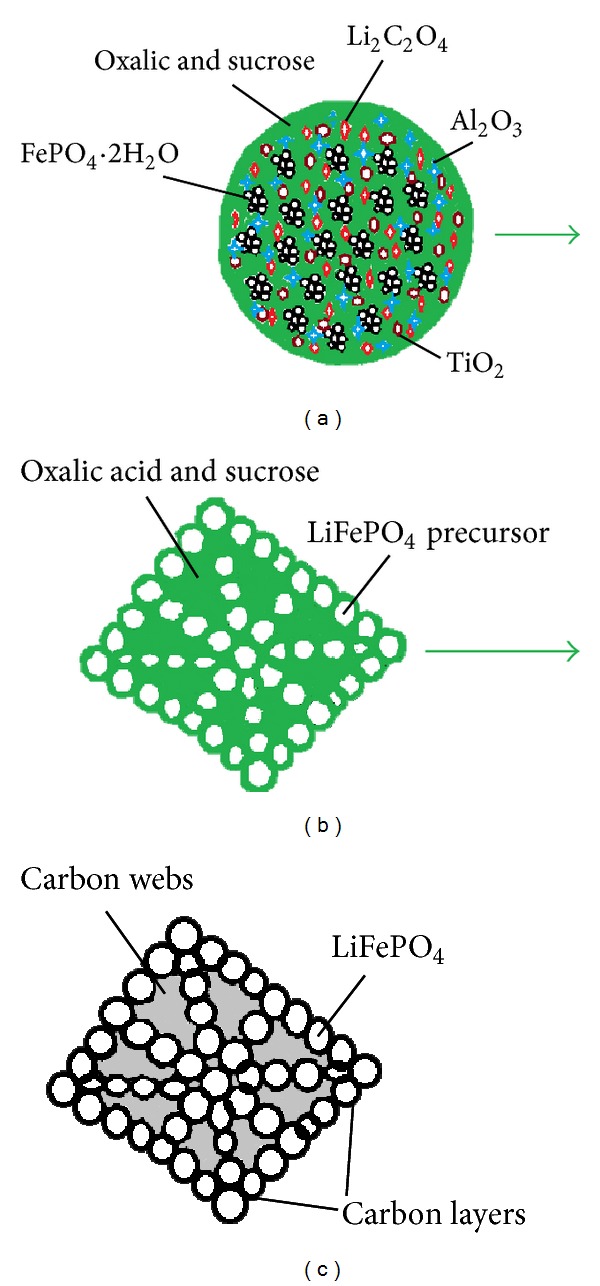
Schematic diagram of spherical LiFePO_4_/C synthesized from nanosized spherical FePO_4_·2H_2_O (a) mixing process, (b) presintering stage and (c) sintered product.

Nanosized spherical FePO_4_·2H_2_O has been used to prepare LiFePO_4_/C. As seen in Figure 1, the SEM images of LiFePO_4_/C from nanosized spherical FePO_4_·2H_2_O were shown. The products had diverse morphology. The as-prepared LiFePO_4_/C composite was spherical and the particles' size close to 100 nm in size was uniformly distributed. The shapes of particles were traced back, following each step of preparation (Figure 2(a) for mixture, Figure 2(b) for presintering product, and Figure 2(c) for sintered product). Oxalic acid as reductant was ultimately decomposed into CO_2_ and H_2_O. Sucrose as carbon source was used for coating nanosized LiFePO_4_ particles.

**Figure 3:**
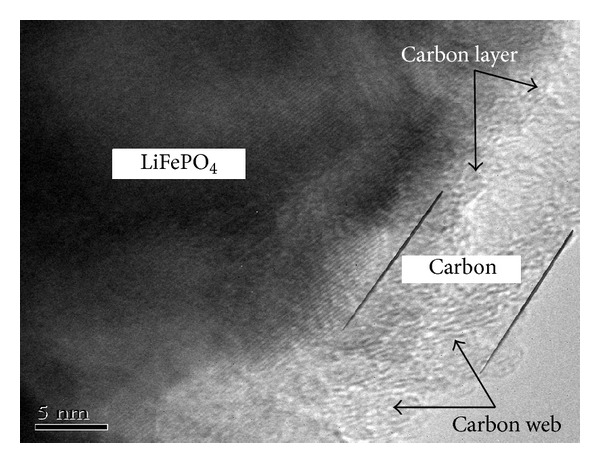
TEM image of spherical LiFePO_4_/C synthesized from nanosized spherical FePO_4_·2H_2_O.

Figure 3 showed the TEM images of LiFePO_4_/C. As shown in the image, LiFePO_4_ particles were well surrounded by a thin surface layer of carbon. The thickness of the carbon-coated layer was about 4 nm. There was a layer of carbon web, providing good electronic contact between LiFePO_4_ particles.

**Figure 4:**
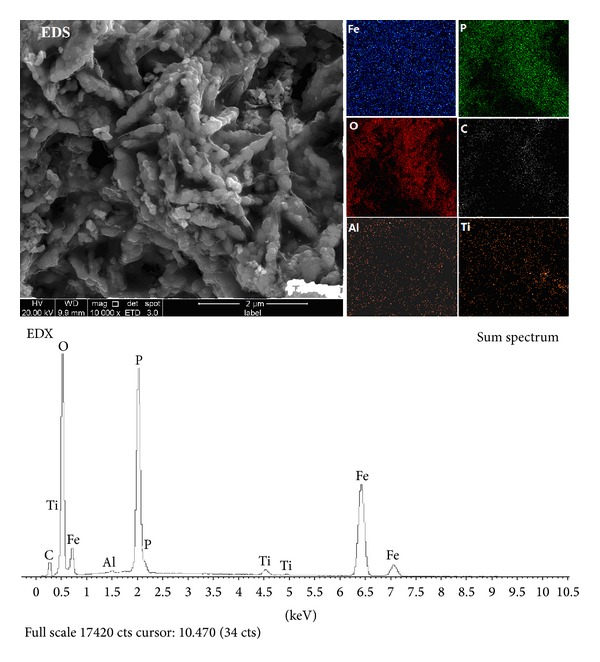
EDS and EDX images of spherical LiFePO_4_/C synthesized from spherical FePO_4_·2H_2_O.

As seen from Figure 4, the EDS and EDX results showed that as-prepared sample had the elements, such as Fe, P, O, C, Ti, and Al, and all of the elements were distributed uniformly, indicating that C, Ti, and Al dispersed in LiFePO_4_ evenly.

### 3.2. Electrochemical Performance of LiFePO_**4**_/C Prepared with Nanosized Spherical FePO_**4**_·2H_**2**_O 

#### 3.2.1. Initial Charge/Discharge Curves and Cycling Performance

**Figure 5:**
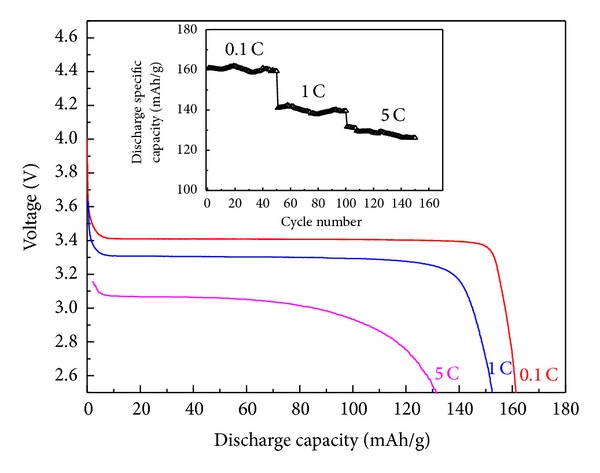
The initial charge/discharge curves and cycle performance.

The theoretical capacity of stoichiometric LiFePO_4_ is 170 mAh·g^−1^.  Figure 5 showed the initial charge-discharge capacity and cycling performance of the LiFePO_4_/C composite cathodes at three different rates of 0.1 C, 1 C, and 5 C. The discharge capacity of LiFePO_4_/C composite synthesized with nanosized spherical FePO_4_·2H_2_O was 161.7, 152.4 and 131.7 mAh/g at 0.1 C, 1 C and 5 C, respectively. Noticeably, the long and flat voltage plateaus were at 3.44 and 3.40 V at the rate of 0.1 C for Li extraction and insertion, respectively. It implied the excellent two-phase redox reaction between FePO_4_ and LiFePO_4_, as well as the typical electrochemical features of olivine-type LiFePO_4_. The small voltage difference between the charge and discharge plateaus at about 0.04 V was representative of good kinetics, especially considering the poor electronic conductivity and low electrochemical diffusion rate of Li ions in a solid phase.

The cycle life of LiFePO_4_/C composite synthesized from nanosized spherical FePO_4_·2H_2_O at the rates of 0.1 C, 1 C, and 5 C was shown. After 50 cycles, the capacity retention obtained 99.1%, 98.7%, and 95.8%, respectively.

#### 3.2.2. High and Low Temperature Performance

**Figure 6:**
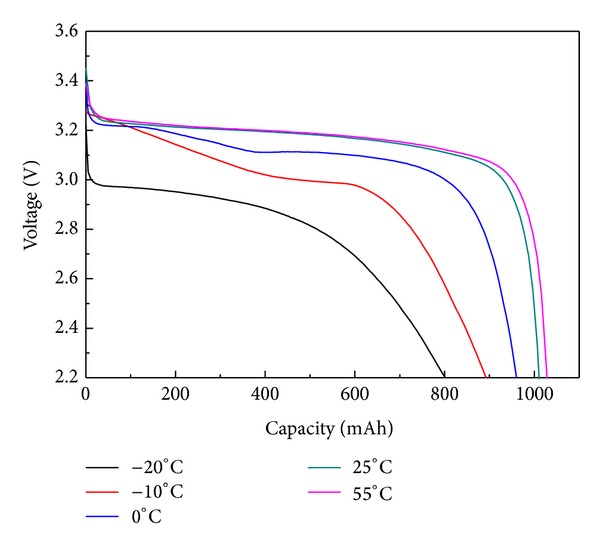
The high and low temperature performance.

The fatal disadvantage of commercial LiFePO_4_ is that it is poor at low temperature performance. The general low temperature was referred to −20°C. Over a voltage range of 2.0 V to 3.65 V, commercial LiFePO_4_ had a capacity retention ratio of 60%~70% at 0.5 C at −20°C by comparison with that of room temperature (25°C). Therefore, to resolve the problem plays an important role in industrialized application. The performance of LiFePO_4_ can be characterized by 18650 battery with designed capacity of 1000 mAh. As illustrated in Figure 6, the high and low temperature performance of the LiFePO_4_/C composite cathodes delivered the outstanding progress to the rate of 0.5 C over a voltage range of 2.2 V to 3.6 V for 18650 battery. Meanwhile, it was found that the battery exhibited a discharge of 1028.3 mAh, 1010.9 mAh, 960.4 mAh, 892.9 mAh, and 801.5 mAh at 55°C, 25°C, 0°C, −10°C, and −20°C, respectively. Compared with discharge performance at room temperature (25°C), the capacity retention maintained 101.7%, 95.0%, 88.3%, and 79.3% at 55°C, 0°C, −10°C, and −20°C, respectively. Moreover, the high discharge voltage plateaus at various temperatures demonstrated the as-prepared sample to be the excellent material for industrialized application.

#### 3.2.3. CV and EIS

**Figure 7:**
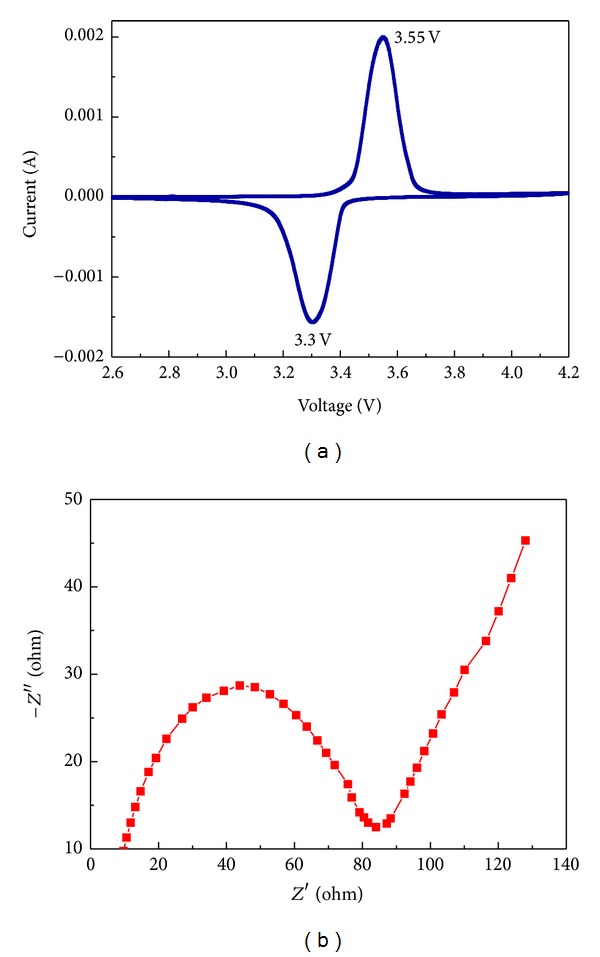
The cyclic voltammogram curves and EIS plots.

Freshly deposited LiFePO_4_/C composite has been examined by CV at a scan rate of 0.1 mV·s^−1^, as shown in Figure 7.  Figure 7(a) showed that the first CV curve of the LiFePO_4_/C composite synthesized with nanosized spherical FePO_4_·2H_2_O was described. The voltage difference was 0.19 V. The voltage observed at 3.52 V in the anodic sweep and at 3.33 V in the cathodic sweep characterized the extraction and insertion of Li^+^ in the LiFePO_4_ olivine structure, respectively.

Meanwhile, as we can seen from Figure 7(b), the electrochemical impedance of LiFePO_4_/C synthesized from nanosized spherical FePO_4_·2H_2_O was shown. The curve was formed by a depressed semicircle in the high- to middle-frequency region and a straight line in the low-frequency range. According to the literature [39], the depressed semicircle represented the charge-transfer reaction between the active materials and the electrolyte (*R*_ct_). From the result, *R*_ct_ had the charge-transfer reaction resistance of 82 Ω, indicating the excellent electrical conductivity and Li-ion diffusion of LiFePO_4_/C composites.

## 4. Conclusions

LiFePO_4_/C composites were then prepared from nanosized spherical FePO_4_·2H_2_O, Li_2_C_2_O_4_, aluminum oxide, titanium oxide, oxalic acid, and sucrose by binary sintering process. The EDS and EDX showed that Ti and Al were distributed in LiFePO_4_/C uniformly. And the composites had good conductive carbon webs and delivered an initial discharge capacity of 161.7 mAh·g^−1^ at a 0.1 C rate, 152.4 mAh·g^−1^ at a 1 C rate, and 131.7 mAh·g^−1^ at a 5 C rate. And the capacity retention obtained 99.1%, 98.7%, and 95.8%, respectively, after 50 cycles. Meanwhile, the high and low temperature performance is excellent for 18650 battery, exhibiting a discharge of 1028.3 mAh, 1010.9 mAh, 960.4 mAh, 892.9 mAh, and 801.5 mAh at 55°C, 25°C, 0°C, −10°C, and −20°C, respectively.

In summary, the as-prepared LiFePO_4_/C materials had favorable properties for their commercial applications.
